# Step count as a digital mobility outcome in orthopedics and orthopedic trauma surgery: a scoping review

**DOI:** 10.1530/EOR-2025-0165

**Published:** 2026-02-04

**Authors:** Benedikt J Braun, Bernd Grimm, Meir T Marmor, Georg Osterhoff, David A Back, Maximilian M Menger, Carolina Vogel, Tina Histing, Dannik Haas, AO Trauma Digital Expert Group, Digital Working Group of the DGOU

**Affiliations:** ^1^ University Hospital Tuebingen, Eberhard-Karls-University Tuebingen, BG Unfallklinik, Tuebingen, Germany; ^2^ Human Motion, Orthopaedics, Sports Medicine and Digital Methods Group, Institute of Health, Transversal Activities, Strassen, Luxembourg; ^3^ Orthopaedic Trauma Institute (OTI), San Francisco General Hospital, University of California, San Francisco, California, USA; ^4^ Department of Trauma and Orthopaedic Surgery, BG Klinikum Unfallkrankenhaus Berlin, Berlin, Germany; ^5^ Center for Musculoskeletal Surgery, Charité-Universitätsmedizin Berlin, Corporate Member of Freie Universität Berlin, Humboldt-Universität zu Berlin, and Berlin Institute of Health, Berlin, Germany

**Keywords:** wearable device, smartphone, activity assessment, fracture, outcome measurement, digital mobility outcome

## Abstract

The need to collect objective outcome parameters digitally is increasing in both clinical practice and research. Step count is a frequently utilized digital mobility outcome (DMO) in orthopedic traumatology; however, its usefulness to monitor the patient recovery process remains unclear. The aim of this scoping review is to investigate the application and utility of daily patient step count as a DMO in musculoskeletal injuries.
PubMed and consensus.app were queried. Eligibility criteria included the following: articles published within 20 years including patients with orthopedic trauma conditions and utilizing daily step count as an outcome. The type of study, case numbers, conditions investigated, use/usefulness of step count, duration of assessment, sensor use and location, and data harvesting specifics were assessed.
Totally, 40 articles were analyzed, revealing an increasing trend in annual publications. The majority of studies were observational (93%), with a mean of 103 participants (range: 9–666). Proximal femur fractures (*n* = 7), anterior curciate ligament (ACL) injuries (*n* = 6), and joint replacement (*n* = 5) were the most frequently investigated conditions. Overall, 30% of studies used step count to demonstrate an association with patient-reported outcome measures, while 27% employed it to identify differences between study groups. Research-grade accelerometers/inertial measurement units (73%) were the most common sensors, with continuous measurement durations from 4 to 14 days.
This review indicates an increasing use of step count as an objective DMO in the orthopedic trauma surgery literature. However, the implementation, application, setup, and data acquisition methodologies remain underexplored. This review highlights current trends and identifies key areas requiring further investigation in future research.

## Introduction

Digital mobility outcomes (DMOs) refer to the measures of a person’s physical movement and walking behavior in real-life, unsupervised settings, typically using wearable sensors or other digital technologies. The integration of DMOs obtained from wearable systems is gaining increasing interest in both research and clinical practice in orthopedic trauma care. This is driven by the premise that these systems offer a way to objectively and continuously monitor the patient recovery pathway. A recent survey of AO (Arbeitsgemeinschaft Osteosynthesefragen) trauma surgeons highlighted this trend, revealing that a significant proportion of surgeons are already utilizing wearable systems to track patient progress in real-world settings (1). Here, accelerometry (through a dedicated device or smartphone) has emerged as one of the most prevalent wearable technologies, favored for its ability to capture patient’s general physical activity patterns throughout the treatment process (2). This trend is further highlighted by a comprehensive review conducted by Marmor *et al.*, which confirmed the increasing use of wearable activity monitors in orthopedic trauma surgery over the past decade (3). This review was meant to emphasize the widespread use of accelerometry, with step count and activity time identified as the key metrics for quantifying patient activity. Accordingly, recent studies have shown the principal capacity of the daily patient step count to track the postoperative recovery process of patients after an extremity fracture (4, 5). However, questions of the clinical value of daily step count to track recovery in different injuries, conditions, and populations and those of the technology to assess this parameter persist and a true standard has yet to be defined. As the field progresses, it is crucial to address these gaps in knowledge. The aim of this scoping review is to identify and map the use of daily patient step count as an outcome measure in musculoskeletal injury studies and, furthermore, to discuss research gaps regarding the usefulness of daily patient step count as an outcome measure in musculoskeletal injuries.

## Methods

### Setting and search

This scoping review was set up to map the available literature on the use of daily patient step count as an outcome measure in musculoskeletal injury studies according to the PRISMA guidelines for scoping reviews (PRIMSA-SCR) (6). The electronic database of PubMed/MEDLINE along with consensus.app was queried to identify relevant studies. The search combined medical subject headings (MeSH) terms and keywords related to musculoskeletal injuries and activity monitoring, specifically focusing on step count. The exact search string used was as follows: ((“musculoskeletal system”(mesh) AND “wounds and injuries”(mesh)) OR (“musculoskeletal injury” OR “musculoskeletal injuries” OR “orthopedic injury” OR “bone injury” OR “soft tissue injury” OR “fracture”)) AND (“step count” OR “steps per day” OR “daily steps” OR “walking activity” OR “activity monitoring” OR “activity tracker” OR “pedometer” OR “accelerometer” OR “ambulatory monitoring”(mesh) OR “walking”(mesh)) NOT (“review”(publication type) OR “systematic review”(publication type) OR “case reports”(publication type)).

This search was conducted on November 3, 2024. The initial search yielded 2,503 abstracts for screening. Following the database search, all identified records were imported to a reference management online repository (rayyan.ai) for collaborative duplicate identification and abstract and full text screening. All titles and abstracts were independently screened by two reviewers (DH and BJB) based on the predefined inclusion and exclusion criteria below. Any disagreements regarding inclusion were resolved through discussion between the two reviewers or by consultation with a third reviewer (CV) if consensus could not be reached.

### Inclusion criteria

Inclusion criteria were as follows: studies involving patients diagnosed with musculoskeletal injuries or conditions relevant to orthopedics and trauma surgery. This includes, but not limited to, studies focusing on fractures, soft tissue injuries, ligamentous injuries, or post-injury rehabilitation and recovery specifically in this population and studies that specifically report or use step count (or equivalent objective terms such as steps per day, daily steps, or walking activity) as a primary or secondary outcome measure. The step count data must be obtained through objective measurement methods (e.g. activity monitors, pedometers, accelerometers, and wearable sensors) but not self-reported or estimated without device-based monitoring. Studies of quantitative research designs, including observational studies (e.g. cohort studies and case–control studies), interventional studies (e.g. randomized controlled trials and non-randomized trials), and clinical trials, were included. Studies published in English were included. Studies published within the last 20 years from the date of the search were included to capture recent research and technological advancements in activity monitoring.

### Exclusion criteria

Exclusion criteria were as follows: studies focused exclusively on healthy individuals without a history of musculoskeletal injury or trauma relevant to orthopedics/trauma. Studies involving populations with non-musculoskeletal conditions that are not directly related to a musculoskeletal injury (e.g. studies solely on neurological conditions, cardiovascular diseases, general geriatric populations, or pediatric populations without a musculoskeletal injury focus) were excluded. Studies where step count or an equivalent objective measure is not reported or used as an outcome measure were excluded. Studies where step count is only mentioned incidentally, is self-reported, or is estimated without the use of objective digital monitoring devices were excluded. Qualitative studies, systematic reviews, meta-analyses, literature reviews, editorials, letters, expert opinions, or case reports that do not present original quantitative data on step count in the defined population were excluded.

### Data analysis

Abstracts deemed potentially relevant by at least one reviewer were retrieved in full text. The full-text articles were then independently assessed against the same inclusion and exclusion criteria by the two reviewers. A standardized data extraction form based on the review questions and objectives was used. The following data were extracted: study characteristics (e.g. title, publication year, country, and study design), participant characteristics (number and specific musculoskeletal injury or condition studied), details of the step count measurement method (e.g. device type, wear location, wear duration, and data harvesting/processing), how step count was reported, and its primary use and key findings related to step count as an outcome. The extracted data were synthesized to provide a descriptive summary of the included studies with the aim to provide an overview of the existing evidence base and to identify potential gaps in the literature.

## Results

### Screening characteristics

**Figure 1:**
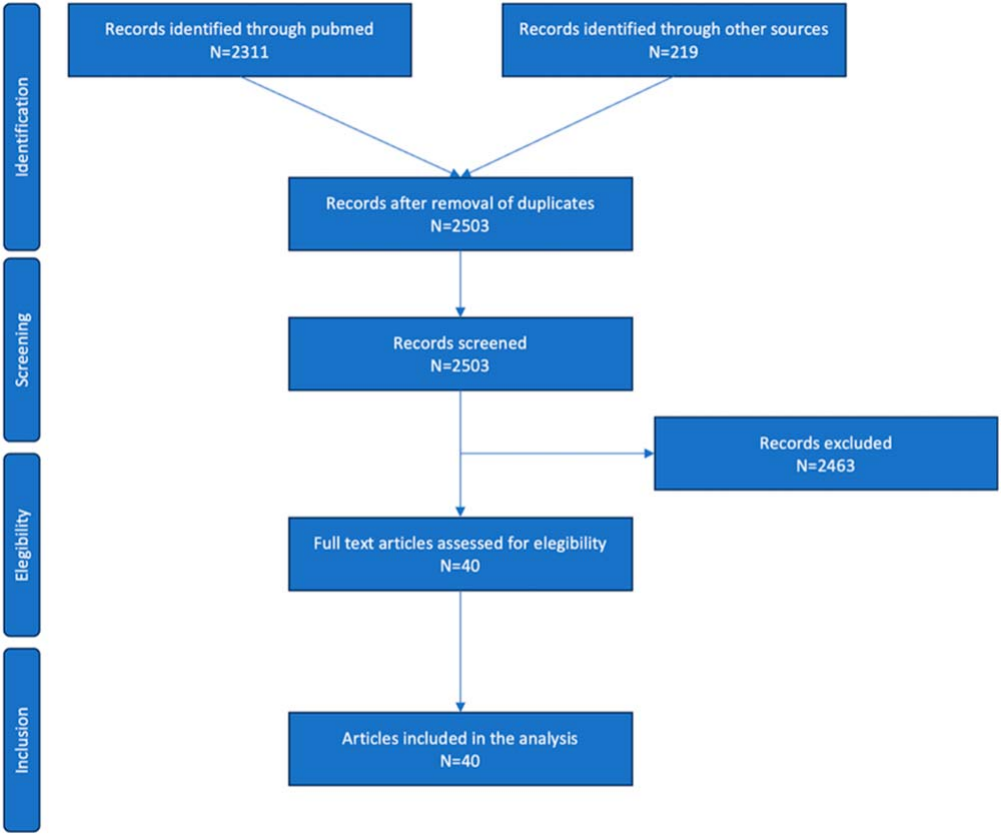
Study flow diagram from identification and screening to eligibility and final inclusion.

The initial search yielded 2,311 abstracts identified through PubMed/MEDLINE and another 219 identified through consensus.app. Following the removal of duplicates, 2,503 unique records were screened at the title and abstract level based on the predefined inclusion and exclusion criteria. Of these, 40 abstracts were deemed potentially relevant and retrieved for full-text review. After full-text assessment, 40 studies met all inclusion criteria and were included in this scoping review. The study selection process is detailed in the flow diagram (Fig. 1).

### Publication and demographic characteristics

**Figure 2:**
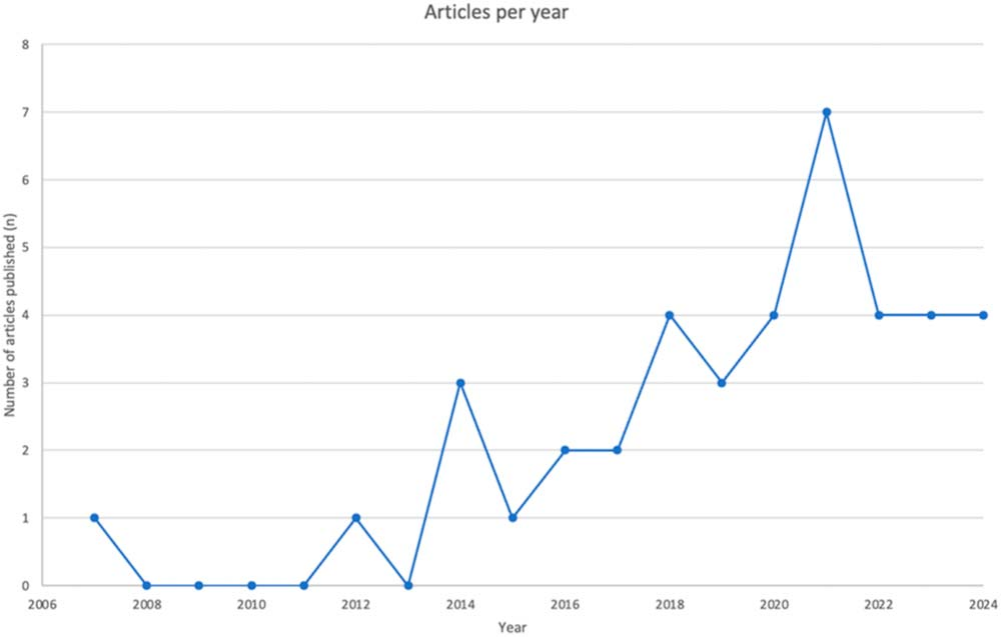
The number of articles published (y-axis, *n* of articles published) per year (x-axis, year of publication) is shown from 2007 to 2024.

The included studies were published between 2007 and 2024. Analysis of publication dates showed an increasing trend in the number of studies published per year over the review period (Fig. 2). The included studies originated from various countries across the globe. The top three countries contributing to the literature were USA (*n* = 15, 37.5%), Australia (*n* = 8, 20%), and Germany (*n* = 6, 15%). The remaining studies originated from Japan (*n* = 4, 10%); Canada (*n* = 2, 5%); and the UK, Hong Kong, Sweden, Denmark, and the Netherlands (each *n* = 1, 2.5%). Analysis of study designs revealed that observational studies were the most common design employed, accounting for 92.5% of the included studies (*n* = 37), with the rest being interventional in design (*n* = 3, 7.5%). Studies investigated, on average, 81 participants concerning the average daily step count and continuously monitored the daily step count over a range of 9–666 days (Table 1). The studies investigated a range of musculoskeletal injuries and conditions relevant to orthopedics and trauma surgery. The majority of studies focused on injuries affecting the lower extremities (*n* = 30, 75%), including proximal femur fractures (*n* = 7), ACL injuries (*n* = 6), joint replacement of the hip and knee (*n* = 7), foot and ankle surgeries (*n* = 4), lower limb amputation (*n* = 4), and a mixed inclusion of lower extremity orthopedic injuries and fractures (*n* = 2). Overall, four studies (10%) focused on both upper and lower extremity injuries, while two studies each (5%) concentrated on spinal injuries and osteoporosis, and one study each on isolated upper extremity conditions and pelvic fractures (2.5%, each).

**Table 1:** Overview of the included studies.

Study	Year published	Patients, *n*	Observed injury/fracture	Sensor location	Step count		Duration assessed, days
					Primary use	Use achieved	
Barchek *et al.* (26)	2021	19	ACL injury	Wrist	Association with PRO	No	4–14
Braun *et al.* (4)	2023	48	Any MSKI	Multiple*	Visualize patient recovery journey	Yes	2–3 mo
Braun *et al.* (16)	2024	38	Any MSKI	Multiple*	Association with activity/gait parameters	Yes	2–3 mo
Capone *et al.* (20)	2021	94	F&A surgery	Hip	Association with lab work	Yes	4–14
Chu *et al.* (50)	2016	21	LLA	On prosthesis	Differentiate between patient groups	Yes	2–3 mo
Crizer *et al.* (35)	2017	589	HJR + KJR	Smartphone on body	Association with PRO	Yes	4 mo–1 y
Davenport *et al.* (27)	2015	20	PFF	NS	Association with physical therapy demand/discharge to home	No	4–14
Davis-Wilson *et al.* (28)	2022	31	ACL injury	Hip	Association with lab work	Yes	4–14
Davis-Wilson *et al.* (21)	2022	66	ACL injury	Hip	Association with PRO	No	4–14
Ekegren *et al.* (14)	2021	63	U/LEF	Thigh (activPAL); hip (ActiGraph)	Differentiate between patient groups	Yes	4–14
Ekegren *et al.* (15)	2020	83	U/LEF	Thigh (activPAL); hip (ActiGraph)	Association with activity/gait parameters	Yes	4–14
Fleig *et al.* (32)	2016	49	PFF	Waist	Association with PRO	No	4–14
Gaffney *et al.* (49)	2024	14	LLA	Thigh	Differentiate between patient groups	Yes	2–4 wk
Ghaffari *et al.* (22)	2023	21	Lower extremity MSKI	Thigh	Association with activity/gait parameters	Yes	1–2 mo
Gilmore *et al.* (12)	2020	216	DSS	Thigh	Association with PRO	Yes	4–14
Hahn *et al.* (31)	2012	18	F&A surgery	NS	Visualize patient recovery journey	No	4–14
Höll *et al.* (23)	2018	46	HJR	Ankle	Visualize patient recovery journey	Yes	2–4 wk
Hubbard-Turner *et al.* (47)	2015	20	F&A surgery	Hip	Differentiate between patient groups	Yes	4–14
Jelsma *et al.* (36)	2021	32	HJR	Thigh	Association with PRO	Yes	4–14
Keppler *et al.* (52)	2020	39	PFF	Wrist	Differentiate between patient groups	Yes	4–14
Kuenze *et al.* (29)	2021	12	ACL injury	Wrist	Target parameter for intervention	Yes	1–2 mo
Ledoux *et al.* (37)	2018	234	F&A surgery	NS	Association with PRO	Yes	NS
Lisee *et al.* (24)	2022	36	ACL injury	Hip	Association with activity/gait parameters	Yes	4–14
Master *et al.* (10)	2021	212	DSS	Hip	Association with PRO	Yes	4–14
Mendel *et al.* (11)	2021	37	Pelvic fractures	NS	Differentiate between patient groups	Yes	4–14
Nakashima & Kukihara (38)	2020	40	HJR	Shoulder	Association with PRO	Yes	4–14
Noeske *et al.* (39)	2025	216	PFF	Thigh	Association with PRO	Yes	4–14
North *et al.* (40)	2023	42	LEF	Insole	Association with PRO	Yes	2–4 wk
O`Halloran *et al.* (34)	2016	30	PFF	NS	Target parameter for intervention	Yes	4–14
Orsi *et al.* (30)	2024	666	HJR	Smartphone on body	Association with PRO	Yes	2–3 mo
Park *et al.* (19)	2007	172	Osteoporosis	Waist	Association with bone density	Yes	≥1 y
Reppas‐Rindlisbacher *et al.* (25)	2021	62	PFF	Wrist	Visualize patient recovery journey	Yes	4–14
Rickert *et al.* (13)	2024	40	Shoulder surgery	Wrist	Visualize patient recovery journey	Yes	2–4 wk
Segal *et al.* (45)	2014	10	LLA	On prosthesis	Differentiate between patient groups	No	4–14
Shephard *et al.* (18)	2017	496	Osteoporosis	Waist	Association with bone density	Yes	≥1 y
Sherman *et al.* (51)	2019	9	LLA	On prosthesis	Differentiate between patient groups	Yes	1–2 mo
Shiomoto *et al.* (48)	2023	60	HJR	Thigh	Differentiate between patient groups		
Taylor *et al.* (33)	2021	57	PFF	Thigh	Association with activity/gait parameters	No	4–14
Triplett *et al.* (46)	2021	10	ACL injury	Waist	Differentiate between patient groups	Yes	4–14
Van der Walt *et al.* (41)	2018	163	HJR + KJR	Wrist	Differentiate between patient groups	Yes	2–3 mo

DSS, degenerative spine surgery; MSKI, musculoskeletal injury; F&A, foot & ankle; HJR, hip joint replacement; KJR, knee joint replacement; LEF, lower extremity fractures; LLA, lower limb amputation; mo, months; NS, not specified; PFF, proximal femur fractures; U/LEF, upper/lower extremity fractures; wk, weeks; y, year.

* Multiple systems used with potential for different sensor placements.

### Use of step count as an outcome measure

The included studies primarily utilized the daily step count in several ways to assess activity, recovery, or the effects of interventions in patients with musculoskeletal injuries. Overall, 12 studies (30%) explored the association between step count and various patient-reported outcomes (PROs), such as pain, function, quality of life, or disability scores. Among these studies, eight reported a statistically significant or clinically relevant association between step count and PROs. Furthermore, step count was also assessed in relation to other objective or clinical parameters in ten studies (25%). These parameters included other activity and gait parameters, bloodwork, bone density, and physical therapy demand/return to home status. Of these, eight studies successfully found a significant association between step count and these parameters. Another 11 studies (27.5%) utilized the daily average step count to compare activity levels between different groups of patients (e.g. different injury types, different treatment methods, and different recovery stages). Of these comparative studies, eight reported significant differences in step count between the groups being compared. Two studies (5%) incorporated step count as part of an intervention or to monitor the effectiveness of an intervention (e.g. rehabilitation programs, specific exercises, and educational strategies aimed at increasing activity). Among the studies evaluating step-based interventions, all reported a positive outcome related to step count or other related measures. Another five studies (12.5%) utilized step count data to visualize or track the trajectory of recovery over time in a descriptive fashion. This entailed monitoring changes in daily step count throughout different phases of rehabilitation. Including these studies and two more that failed to show other associations, seven successfully demonstrated or primarily utilized the visualization of increasing step count as an indicator of recovery progression. Of the studies that included a pre-op step count, all but one looking at ankle arthrodesis and arthroplasty with intermediate follow-up in 18 patients were able to show a characteristic recovery profile with a postoperative decrease in step count and subsequent increase.

### Characteristics of wearable device use

**Figure 3:**
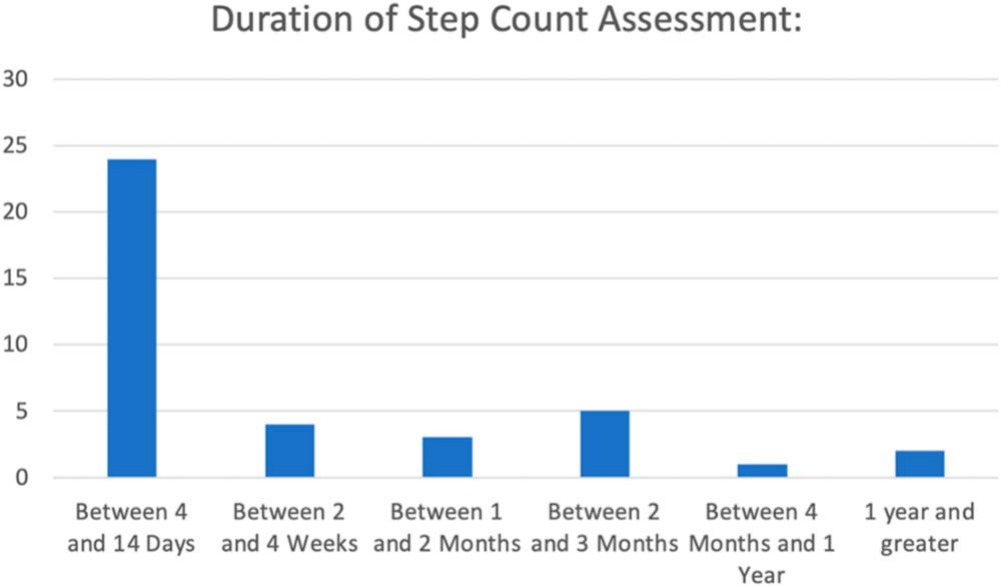
The distribution of articles (y-axis, *n* of articles published) and their respective wearable measurement time (y-axis, categorized measurement time) are shown.

Overall, the studies employed a variety of wearable devices and methodologies to capture step count data. The duration for which participants were instructed or monitored to wear the activity tracking devices varied across studies. The reported durations of continuous daily step count recording ranged from 4 to 14 days to over 1 year of assessment (Fig. 3).

**Figure 4:**
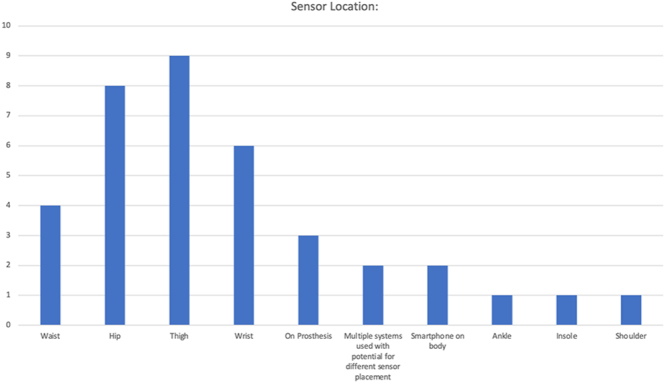
The distribution of articles (y-axis, *n* of articles published) and their respective wearable placements on the body (y-axis, placement type of wearable system) are shown.

Of the 40 articles, ten (25%) used the daily step count collected prior to an injury/intervention as a frame of reference for the post-injury/intervention recovery process. A range of activity monitoring systems and devices were used, reflecting the technological landscape over the review period. These included research-grade activity monitors (inertial measurement unit/accelerometer) in 29 articles (72.5%), consumer-grade wearables (e.g. smartwatches and fitness trackers) in five articles (12.5%), patient-owned systems/smartphones in four articles (10%), and plantar pressure insoles in one article; one article did not specify the system. The location of the sensor on the body varied between studies and included waist, hip/thigh, wrist, on prosthesis, ankle, shoulder, insole, and multiple placement options. The distribution is shown in Fig. 4. Five articles did not specify the sensor placement. The methods for collecting and processing data from the wearable devices also varied and were influenced by limited information provided. The most common approach was using system-specific software for harvesting and data curation (*n* = 19, 47.5%), with another 11 (27.5%) not reporting but using systems that generally only allow for system-specific software use. In total, six authors (15%) stated that they manually exported and curated data without using system-specific software, with another two authors (5%) not reporting but using systems that generally only allow for manual export. Finally, two authors (5%) did not specify the approach and no inferences could be made.

## Discussion

This scoping review investigated the application and utility of individualized daily patient step count assessment as a DMO in musculoskeletal injuries, revealing clear trends and areas for future exploration. The increasing distribution of consumer-grade wearable systems, including smartphones in the general population, is mirrored by the increasing number of publications using these technologies in orthopedic and trauma surgery research over the past two decades, indicating a rising interest in objective, wearable-based tracking of the patient recovery process. This general trend is confirmed by the overall rise in digital outcome assessment across various medical specialties (7, 8), with a high overlap and potential cross-discipline use also concerning the orthopedic trauma surgery (9). Despite the increasing number of systems and publications, our scoping review highlights that research in this area is still in its infancy. The majority of identified studies were observational in nature (92.5%), often lacking comparator groups, suggesting that while step count is being explored, the evidence base is largely descriptive, reflecting an early phase of technological adoption and outcome validation including the lack of any recognized general, disease-specific or recovery-state-specific reference values and less so, longitudinal trajectory profiles characteristic of distinct disease progression, recovery pathways or specific events.

### Use and utility of step count

In our scoping review on the daily use of step count, predictably, the majority of studies focused on lower extremity injuries (75%), where the link between injury and step count is most direct. However, step count was explored in both spinal and pelvic ring injuries and also in studies including also upper extremity fractures and orthopedic conditions (4, 10, 11, 12, 13, 14, 15, 16). While less intuitive for upper extremity cases, pain interference can significantly impact overall mobility and, consequently, step count also in the upper extremity (17). This highlights that step count can potentially also serve as a high-level indicator of overall function and activity even when the primary injury is not in the lower limbs. The applied use cases for step count in the literature are diverse, with a generally high level of reported utility of using step count as an outcome. Step count appears useful for tracking various patient-reported outcomes (PROs) and parameters associated with activity, such as bone density (18, 19) or even certain lab values (20, 21). At the most rudimentary level, the base value of a daily step count tracking over time in the observed literature is visualizing its progression over time. This has been shown by several authors to be able to visualize a generally increasing trend in recovering patients after an injury/intervention (4, 11, 13, 22, 23, 24, 25). The injuries already studied, which are amenable to it, are both musculoskeletal injuries in the upper and lower extremity, as well as more from the orthopedic spectrum with joint replacements and also ACL reconstruction (26, 27, 28, 29, 30, 31, 32, 33, 34). This represents the most basic level of recovery tracking through patient gait activity just in a descriptive fashion. Added value can be obtained, when patient-owned wearable systems are used, that have been active prior to an injury, or when a dedicated system is used in a degenerative case applied prior to an intervention. This allows for better categorization and interpretation of the postoperative recovery trajectory in relation to the pre-injury/pre-intervention activity, showing true recovery to a pre-injury state of function or habitual behavior, or improvement from a previously low activity situation. In the context of trauma surgery with patient-owned systems, this approach has been labeled the ‘bring your own device’ approach (4). Going one step further than just visualizing the parameter are several works showing the association that step count has with various patient-reported outcomes (PROs) and other objective outcomes. In this review, we have identified literature showing an association with PROs, such as the lower extremity function score, hip disability and osteoarthritis outcome score (HOOS), Short Form 36 (SF-36), or the University of California Los Angeles (UCLA) score (10, 35, 36, 37, 38, 39, 40). Furthermore, associations between daily step count and socioeconomically relevant parameters, such as return to independent mobility, health status, and return to work, were reported by several authors (12, 14, 16), showing higher daily step count with improvements in these domains. Finally, physiological effects in relation to the daily step count with a change in peritendinous lactate/pyruvate levels after Achilles tendon rupture and cartilage oligomeric matrix protein in ACL reconstruction, as well as changes in bone density associated with different step count levels, were described by different authors (18, 19, 20, 21). Furthermore, depending on the observed injury, step count can serve as an outcome and an interventional measure as step-based feedback training has been shown to have benefits over non-feedback training. In a study by Van der Walt *et al.*, subjects receiving step-based feedback after hip and knee arthroplasty had a significantly higher mean daily step count early (weeks 1–6) and late during the recovery process (6 months) compared to patients that did not receive feedback. Subjects receiving feedback training were 1.7 times more likely to achieve a mean 7,000 steps per day, and 70% of subjects had a greater mean daily step count compared with their preoperative level at 6 months (41). This study also shows an apparent benefit of daily step count as a DMO because it is an easily understandable and actionable measure for patients, something other DMOs including those related to walking such as walking speed or asymmetry may not be able to offer. These applications are consistent with the known broader uses of step count and other wearable-related metrics beyond daily tracking, such as assessing fall risk (42, 43). Another use-case of step count lies in patient risk stratification, also for complications associated with higher levels of activity. The group out of Aarhus has shown that the daily physical activity, including the number of steps, of patients who underwent metal-on-metal hip replacement influenced the serum ion concentrations and risk of pseudotumor formation (44). It shows that physical activity can thus serve as a tool to tailor treatment and choice of implant to individual patient activity levels. This ability to differentiate between different patient groups based on step count was also observed in other works from this review (11, 14, 41, 45, 46, 47, 48, 49, 50, 51, 52).

The main use of daily step count has been in general health and stems from times even before it was digitally assessed. Strong correlations between increased daily step count as a main measure of physical activity and the reduction of various disease risks, including cardiovascular, neurological, and oncological conditions or diabetes, have become evident (53, 54, 55, 56). General threshold or target values for health-preserving behavior have become adopted in the general population (e.g. 10,000 steps/day) or have been adapted for specific age groups or conditions. However, in orthopedic and traumatology use cases or literature, the impact of disease or treatment toward disabling or enabling patients to retain such health-preserving activity levels is rarely reported numerically. The effect of musculoskeletal (MSK) disease or its treatment on initiating, progressing, or preventing secondary non-communicable diseases can thus be objectively quantified (4). This contributes to improving patient care beyond the underlying MSK condition (e.g. prevention) and to assessing the broader health economic value of orthopedic and trauma interventions.

While the utility of daily step count was reported for many clinical use cases, the other outcome parameters assessed alongside the step count across studies were often inhomogeneous, and the sample sizes in most studies were relatively small, limiting the generalizability of these findings and indicating the need for a clearly defined standard set of outcome parameters and a reporting structure backed by international professional societies (57). Furthermore, step count alone may not always offer the highest precision for differentiation, some studies reported negative results (no change in step count or association with other parameters studied) or an incomplete picture. Furthermore, step count measured by wrist-worn devices can be limited by the use of walking aids (crutches, frames, and rolling walkers) often in service during the early weeks after lower extremity injury treatment (58). This has to be accounted for both in the interpretation of the literature and in the setup of new studies looking to explore this metric. The effect can be mitigated somewhat by using research-oriented wearables with higher precision over consumer devices, especially in patients with slower gait speeds, or by choosing another sensor location apart from the hip (58). In addition, studies investigating postoperative physical activity and step count have shown that depending on the condition and intervention, as well as measurement time point, differences in physical activity might not be detectable despite a change in patient-reported outcome after intervention (59, 60). These limitations clearly highlight that daily step count may be more of a baseline, surrogate measure and too multifactorial or unspecific for certain use cases and underscore the notion that step count is likely most effective when used in conjunction with other outcome measures as part of a combined, comprehensive assessment (61). In addition, despite step count being in the top five reported digital outcomes across specialties in a large-scale review, the authors come to the same conclusion that before any wide clinical adoption of this parameter, further research on predictive validity, responsiveness, and ecological validity is necessary (62).

### Systems and use specifics

Our results show that the majority of studies use dedicated devices that are handed out at the start of a study, something that is also seen in other fields (9). This is understandable as the distribution of a dedicated device offers comparable and scalable outcome parameters between all participants. While using the above-mentioned ‘bring your own device’ strategy cannot offer complete comparability both because of different available parameters and because of different hardware in different systems, this has the advantage that compliance issues can potentially be reduced while giving you access to pre-injury data (63). Step count is also a parameter that is available from most systems making it a great, baseline parameter, despite reduced precision that is associated with different systems especially from the consumer-grade field. Another great advantage is that by focusing on this parameter and using systems with low energy demands giving long potential measurement times with very limited patient input, we can get a longer, continuous picture of the patient recovery process, as opposed to classical clinical follow-up that only happens episodically in large increments. Considering measuring specifics, such as sensor type, placement, sampling frequency, measurement duration, and additional outcomes to look at, there is too much heterogeneity in the current literature to come to a generalizable conclusion. As other reviews on similar topics have already pointed out, there is a clear need for a better definition of the ideal use cases, as well as predictive validity, responsiveness, and ecological validity (62). The current review does, however, allow some general takeaways from the assessed articles. When looking to employ a dedicated device, several factors need to be accounted for: injury entity (upper vs lower extremity vs spine/pelvis), potential use of walking aids, pairing with other outcomes, i.e., both device-associated and patient-reported outcomes, and finally, measurement time (pre-injury measurement in degenerative conditions for comparative purposes; continuous measurement, especially in fracture cases; and predictive analysis). With these factors in mind, step count and associated DMOs have the potential to visualize the patient recovery after injury and identify patients in need for further therapeutic interventions (64).

### Limitations

This scoping review provides a broad overview of the current literature on the use of step count as a DMO in orthopedic and trauma surgery. However, it is not without limitations. The review was limited to studies published in English and indexed in PubMed and consensus.app, potentially omitting relevant research from other databases or languages. We chose to perform the analysis in the form of a scoping review to provide an initial overview of this diverse field, which can be hard to assess, especially from a clinician standpoint. As such, more strict criteria on reporting of biomarkers and endpoints were not taken into account (65). Due to the inherent nature of scoping reviews and the search strategy employed, including variations in abstract formatting and keyword usage, some relevant work on wearable monitors in orthopedics and trauma was missed with this review. Both our personal knowledge of the field and the comments of the reviewers have confirmed this. This is associated with the strict inclusion criteria relying solely on abstract screening, but necessary to provide overview over the over 2,000 articles screened for this purpose. To provide a balanced picture of the field free from our subjective experience in the field and in compliance with our inclusion criteria, we chose to not include any articles that do not clearly identify the relevant methodology/parameters in the abstract. A selection of the missed articles that are or were made aware to us has been incorporated in the discussion and conclusion section of the manuscript to ensure that little information is lost (36, 44, 59, 60, 66, 67). This limitation clearly highlights the need for standardized reporting criteria and guidelines for studies utilizing wearable technology in clinical outcomes research, particularly in the field of orthopedics and orthopedic trauma. There are several working groups both nationally and internationally already looking to provide uniform criteria for reporting and selecting sensor technology and the associated outcomes, most notably in the valuable work of the Mobilise-D consortium, trying to provide this across different specialties (68). This ongoing work will certainly facilitate broader use and overview in the future. Furthermore, the heterogeneity in study designs, patient populations, wearable devices, and reporting methodologies across the included studies made a quantitative synthesis challenging. The level of detail regarding data harvesting and processing methods was also inconsistent, limiting the depth of analysis in this area.

### The road ahead

A unique advantage of wearable systems is their ability to track activity continuously over long periods, often requiring minimal patient input once a device is in place. Despite this, physician input is still frequently needed, often involving manual data extraction or reliance on specific software. This highlights a potential for future development in remote data sharing and automated analysis to facilitate virtual checkups and streamline data harvesting in a data-safety-compliant manner. Based on the current landscape, several challenges need to be addressed in future research. There is a clear need for larger-scale, comparative studies to establish the efficacy and specific utility of step count as an outcome measure in different orthopedic and trauma populations both as an isolated measure and in the setting of a combined outcome evaluation system. To enhance comparability and the potential for data pooling, meta-analysis, and exploration using machine and deep learning methods, future studies should aim for more standardized reporting of both the technical aspects of the wearable systems used and the specific parameters collected in conjunction with step count. This would allow for greater insight into which system configurations and complementary measures provide the most valuable information. Larger-scale studies with standardized or at least comparable reporting of daily step count and another set of clinical measures and relevant events (e.g. return-to-X and readmission) will also support the effective deployment of artificial intelligence (AI)-driven methods to remotely identify patients for early intervention; personalized therapy adjustments; or sparing patients, healthcare professionals, and the payer the burden of unnecessary visits or diagnosis. AI-driven methods to analyze the step count underlying accelerometry signal may in the near future also provide clinically relevant step count context such as the surface of walking, for instance on ramps, stairs, or uneven ground, which can be more specific to orthopedic and trauma conditions and rehabilitation (69). Ultimately, step count should be viewed as one piece of a larger puzzle in assessing patient recovery and function. Future research should focus on how step count and other step metrics, including step context/surface or focus on specific steps (on stairs, at a certain speed or intensity), integrate with and complement other objective and subjective outcome measures to provide a more comprehensive understanding of the patient’s journey. In addition, the use of different streams of input from wearables, patient-reported outcomes, and also the rapidly evolving field of implantable sensor technology is an area that needs to be explored in the future, as it has the potential to provide the most comprehensive assessment of the individual recovery course (61, 70, 71).

## ICMJE Statement of Interest

B J Braun and B Grimm are consultants to Bios Medical AG.  The authors declare that there is no conflict of interest that could be perceived as prejudicing the impartiality of the work reported.

## Funding Statement

Authors have received travel support by the AO Foundation.
